# Aberrant expression of microRNAs as biomarker for schizophrenia: from acute state to partial remission, and from peripheral blood to cortical tissue

**DOI:** 10.1038/tp.2015.213

**Published:** 2016-01-19

**Authors:** C-Y Lai, S-Y Lee, E Scarr, Y-H Yu, Y-T Lin, C-M Liu, T-J Hwang, M H Hsieh, C-C Liu, Y-L Chien, M Udawela, A S Gibbons, I P Everall, H-G Hwu, B Dean, W J Chen

**Affiliations:** 1 Institute of Epidemiology and Preventive Medicine, College of Public Health, National Taiwan University, Taipei, Taiwan; 2 Center of Genomic Medicine, National Taiwan University, Taipei, Taiwan; 3 The Florey Institute of Neuroscience and Mental Health, Parkville, VIC, Australia; 4 Department of Psychiatry, The University of Melbourne, Parkville, VIC, Australia; 5 Department of Psychiatry, College of Medicine and National Taiwan University Hospital, National Taiwan University, Taipei, Taiwan

## Abstract

Based on our previous finding of a seven-miRNA (hsa-miR-34a, miR-449a, miR-564, miR-432, miR-548d, miR-572 and miR-652) signature as a potential biomarker for schizophrenia, this study aimed to examine if hospitalization could affect expressions of these miRNAs. We compared their expression levels between acute state and partial remission state in people with schizophrenia (*n*=48) using quantitative PCR method. Further, to examine whether the blood and brain show similar expression patterns, the expressions of two miRNAs (hsa-miR-34a and hsa-miR-548d) were examined in the postmortem brain tissue of people with schizophrenia (*n*=25) and controls (*n*=27). The expression level of the seven miRNAs did not alter after ~2 months of hospitalization with significant improvement in clinical symptoms, suggesting the miRNAs could be traits rather than state-dependent markers. The aberrant expression seen in the blood of hsa-miR-34a and hsa-miR-548d were not present in the brain samples, but this does not discount the possibility that the peripheral miRNAs could be clinically useful biomarkers for schizophrenia. Unexpectedly, we found an age-dependent increase in hsa-miR-34a expressions in human cortical (Brodmann area 46 (BA46)) but not subcortical region (caudate putamen). The correlation between hsa-miR-34a expression level in BA46 and age was much stronger in the controls than in the cases, and the corresponding correlation in the blood was only seen in the cases. The association between the miRNA dysregulations, the disease predisposition and aging warrants further investigation. Taken together, this study provides further insight on the candidate peripheral miRNAs as stable biomarkers for the diagnostics of schizophrenia.

## Introduction

It is now recognized that schizophrenia occurs in individuals with a genetic predisposition but frank illness only occurs after exposure to as yet unknown environmental factors.^1^ There is now strong evidence to suggest that the environmental factors trigger the epigenetic mechanisms that change gene expression in the central nervous system (CNS) to bring about the onset of symptoms.^2, 3^ Hence, it is important that we now know that at least 50% of any change in gene expression in the CNS is correlated with changes of expression of the same gene in peripheral tissue,^4^ suggesting that some peripheral markers will reflect changes in brain chemistry. It is also significant that studies have reported changes in gene expression in human brain^5^ and peripheral cells^6, 7, 8^ as a consequence of anti-psychotic treatment in different illness states (that is, acute or remission state). In addition, it has been shown that changes in gene expression in the CNS of people with schizophrenia are related to their duration of illness (DOI) at death.^9, 10, 11^ Hence, it would appear that changes in gene expressions in people with schizophrenia undergo dynamic changes related to anti-psychotic drug treatment and clinical course. Thus, valid peripheral biomarkers would have a number of uses, including facilitating diagnosis, predicting prognosis and evaluating therapeutic response.^12, 13^

MicroRNAs (miRNAs) can be regarded as master regulators of essential cellular processes^14, 15^ and could therefore be affected by disease processes. Profiling of miRNA is increasingly being undertaken in the search for biomarkers in a variety of diseases.^16^ We^17^ and others^18^ have reported altered miRNAs expression levels in peripheral blood mononuclear cells (PBMC) from people with schizophrenia. Furthermore, it has been reported that anti-psychotic drug treatment is associated with changes in miRNA expression in mouse brain,^19^ human cortical tissue,^20^ human plasma^6, 7^ and T-lymphocyte cell lines.^8^ Therefore miRNA levels have the potential to provide valuable information on the clinical and/or treatment status of a person with schizophrenia. However, unlike mRNA,^9, 10, 11^ little is known about the dynamic changes of miRNAs during the different stage of illness (that is, acute or partial remission).

We previously identified a seven-miRNA (hsa-miR-34a, miR-449a, miR-564, miR-432, miR-548d, miR-572 and miR-652) signature as a potential biomarker for schizophrenia.^17^ Of these miRNAs, hsa-miR-34a was most changed in the PBMC of people with schizophrenia, which is significant as it has been shown to be higher in the dorsolateral prefrontal cortex (Brodmann area 46 (BA46))^21^ and plasma^7^ of people with schizophrenia. In addition, downregulation of hsa-miR-432 in PBMC^18^ and cortical hsa-miR-652^22^ have since been reported in people with schizophrenia. Among the seven miRNAs, hsa-miR-449a and hsa-miR-548d have been shown to be relevant to brain development or function. Hsa-miR-449a shows a striking expression pattern during human pre-natal development,^23^ which may be related to ciliogenesis in the ventricular system,^24^ and hsa-miR-548d is involved in regulating the expression of erb-b2 receptor tyrosine kinase 2 (ERBB2), one of the receptors in NRG1–ErbB signaling pathways.^25, 26^ Thus, changes in the levels of expression of either miRNA could have long-term consequences for brain development and function, making it plausible for them to play a role in the etiology of schizophrenia.

Clearly, many questions are still outstanding with regards to the functional roles of miRNAs in the etiology of schizophrenia and their potential value as biomarkers. To begin to address some of these issues, we determined whether: (i) hospitalization could affect human miRNAs expression, and (ii) to see if there were relationships between changes in specific miRNAs in both the blood and brain from people with schizophrenia. We addressed these aims by: (i) measuring levels of miRNA in PBMC from people with schizophrenia in the acute phase in their illness and again in partial remission state; and (ii) measured levels of two candidates, hsa-miR-34a and hsa-miR-548d, in the dorsolateral prefrontal cortex and caudate putamen from people with schizophrenia and a group of non-psychiatric controls.

## Materials and methods

### Cohorts

#### Peripheral sample cohort

For peripheral studies, blood was collected from participants in the Lipid Biology of Schizophrenia and Schizotypal Traits (LIBISS) project at the Department of Psychiatry, the National Taiwan University Hospital, NTUH Yun-Lin Branch, Taipei City Hospital Song-De Branch. A total of 48 people with schizophrenia (aged 23 to 66) and 37 age- and gender-matched healthy volunteers made up of students and the general population were recruited between August 2009 and July 2012. The people diagnosed as having schizophrenia according to the criteria of the Diagnostic and Statistical Manual of Mental Disorders, Fourth Edition (DSM-IV),^27^ were recruited when hospitalized (acute state, T1). Among the people with schizophrenia, only four were at first onset while the others had varying histories of treatment for the illness. Blood was also collected at a follow-up session within 2 months of discharge from hospital (partial remission, T2). The severity of their symptoms was assessed using the Positive and Negative Syndrome Scale (PANSS)^28^ at both T1 and T2. A comprehensive medication history was obtained for each clinical participant and the most recent prescribed dose of anti-psychotic drug converted to a chlorpromazine equivalence dose.^29^ For the healthy volunteers, they were screened to confirm the absence of a current or previous psychiatric history, using the Diagnostic Interview for Genetic Studies (DIGS). Exclusion criteria for our studies were; severe neurological abnormality, prominent substance use problem or mental retardation. The study was approved by the Institutional Review Board of the National Taiwan University Hospital. Each participant provided written informed consent after being given a complete description of the study.

#### Postmortem sample cohort

The cohorts used for the postmortem study were described in detail in a previous study.^9^ Briefly, this study was carried out using dorsolateral prefrontal cortex (BA46) and caudate putamen from 25 people with schizophrenia and 27 age- and gender-matched non-psychiatric controls; the people with schizophrenia consisted of those with short DOI (<7 years, *n*=13) and those with long DOI (>22 years, *n*=12). The final cohorts of people with schizophrenia and controls were not 100% age–gender-matched because some individuals (three cases and one control) were later excluded due to poor RNA quality. However, the distributions of age and gender in the two groups remained similar (see Table 1). Psychiatric diagnoses were made according to DSM-IV criteria by consensus between two psychiatrists and a psychologist after an extensive case history review using a structured diagnostic instrument, the Diagnostic Instrument for Brain Studies.^30^ Following the case history review, DOI was calculated as the time from first hospital admission to death. A comprehensive medication history was obtained for each participant and the most recent prescribed dose of anti-psychotic drugs was converted to a chlorpromazine equivalence dose.^31^ Approval of the study was obtained from the Ethics Committee of the Victorian Institute of Forensic Medicine and the North Western Mental Health Research. All the tissue was obtained from the Victorian branch of the Australian Brain Bank Network.^9^

### RNA extraction, complementary DNA template preparation and quantitative real-time PCR

For peripheral samples, whole blood (~10 ml) was collected from each participant both at baseline and 2-month follow-up using K3-EDTA anticoagulant tubes. Specimens were processed within 3 h of collection to maintain the integrity of the RNA. PBMCs were isolated using Ficoll-Paque PLUS (Fisher Scientific, Pittsburgh, PA, USA), then total RNA was extracted from the PBMCs using TRIzol reagent (Invitrogen, Grand Island, NY, USA) as per manufacturer's instructions. The RNAs from the peripheral samples were shown to be of high quality, with a RNA Integrity Number of >8 (ref. 17) and a mean RNA quality ratio of OD260/280 of 1.94 (s.d.=0.23). The complementary DNAs of the seven miRNAs were analyzed using reverse transcription–PCR (RT–PCR) with TaqMan MicroRNA Reverse Transcription reagents and primers of TaqMan MicroRNA Assays (Life Technologies, Grand Island, NY, USA; primer details given in Supplementary Table 1). Levels of the seven-miRNAs in 5 ng total RNA were measured using real-time qRT–PCR with ABI PRISM 7900 Real-Time PCR System (Life Technologies). RNU48, a small nuclear RNA, was selected as an internal control for the quantification analysis. All assays were performed in duplicate.

For the postmortem samples, total RNA was extracted from ~100 mg of BA46 and caudate putamen from each participant using TRIzol reagent followed by DNase I (Invitrogen) treatment. All cadavers were kept at 4 °C prior to processing and, following collection, CNS tissue was stored at −80 °C. The postmortem interval, brain pH and the RNA Integrity Number value of RNA were reported in Table 1. All the RNA samples were checked for genomic DNA contamination and the RNA quality assessed as described previously.^32^ The complementary DNA of the miRNAs were made using TaqMan MicroRNA RT reagents and specific primers (Life Technologies) for the miRNAs (see Supplementary Table 1). The qRT–PCR for the miRNAs were performed on 5 ng total RNA using Taqman MicroRNA assays in a Bio-Rad iQ 5 Real-Time PCR Detection System (Bio-Rad, Hercules, CA, USA) with RNU48 as an internal control. All assays were performed in triplicate and the average cycle threshold (Ct value) determined.

### Two miRNAs assayed for the postmortem brain tissue

Owing to the limited amount of the brain tissue available for assay, two miRNAs were selected for the expression level measurement on the following rationale: (i) hsa-miR-34a was most changed in people with schizophrenia in our previous findings in PBMC^17^and was expressed in postmortem brain tissue;^21^ and (ii) hsa-miR-548d was the only miRNA that had direct evidence of schizophrenia-associated NRG1–ErbB signaling pathways^25, 26^ in the view point of functions of miRNAs.

### Statistical analysis

Demographic, CNS collection data and pharmacological data, where appropriate, were compared using the Student's *t*-test (numeric) or *χ*^2^-test (non-numeric).

For data from PBMCs, the normalized threshold cycle number ΔCt, in which ΔCt=(average Ct of target miRNA− average Ct of RNU48), were calculated. Undetermined target miRNAs were assigned with terminative cycle number as 40 for the real-time RT–PCR by using RQ manager software v. 1.2.1 (Life Technologies). Relative expression levels of miRNAs were calculated as 2^(−ΔCt)^, which is commonly used in genome-wide profiling studies of miRNAs.^33^ A paired *t*-test was used to compare each participant's expression levels of the seven miRNAs at baseline (T1) and 2-month follow-up (T2). The Student's *t*-test was used to compare the relative expression of the seven miRNAs between inpatients with schizophrenia and controls at T1. Receiver-operating characteristics (ROC) curves were used to evaluate the sensitivity and specificity of the miRNAs in differentiating between all inpatients and controls, with the area under ROC curve (AUC) used to evaluate the overall predictive power.

For data from postmortem samples, the expression level of each miRNA was calculated by its normalized threshold cycle number ΔΔC_t_, in which ΔΔC_t_=[C_t_ (target miRNA)−Ct (target miRNA of calibration sample)]−[C_t_ (reference miRNA)−Ct (reference miRNA of calibration sample)].^34^ For the DOI subgroup analyses in postmortem sample cohort, a multiple linear regression model was used to determine the variance associated with the both disease and DOI on target miRNA expression levels. Age and gender were included in the model as covariates. Adjusted *P*-value for each group comparison was calculated using Tukey's multiple comparison adjustment. For the correlation analysis of age and miRNA expression levels, the data were first checked using the Cook's distance to exclude the high influence points for data analysis and then correlation coefficients were calculated. The interaction analysis of age and disease status was performed using a multiple linear regression of miRNA expression levels on age, disease status and an interaction term between age and disease status.

All the fold changes in this study were calculated by [average of 2^(−ΔCt)^ in cases/ average of 2^(−ΔCt)^ in controls]. All the analyses were performed using software SAS 9.2 (SAS Institute, Cary, NC, USA) and GraphPad Prism 5 (GraphPad, La Jolla, CA, USA).

## Results

### Demographic and pharmacological data of periphery sample cohort

There were no differences in gender and age between people with schizophrenia and controls (Table 1). However, there was a significant difference in years of education (11.3±2.9 years for cases and 17.1±3.5 years for controls, *t*_80_=8.0, *P*<0.0001). The age at onset and DOI for people with schizophrenia were 23.6±7 years and 16.8±10.5 years, respectively. The average chlorpromazine equivalents of anti-psychotic dose were 453.3±276.1 mg per day.

Of the 26 inpatients that had information on the PANSS scale at two time points, there were significant improvements in the total scores (T1=71.1±19.2, T2=61.9±24.1, *t*_24_=3.98, *P*<0.001), positive symptoms (T1=19.4±4.3, T2=16.0±6.8, *t*_24_=3.97, *P*<0.001) and general psychopathology symptoms (T1=33.6±10.4, T2=29.3±10.4, *t*_24_=4.38, *P*<0.001) at discharge in comparison with admission time point (Supplementary Table 2).

### miRNAs of periphery sample cohort

**Figure 1:**
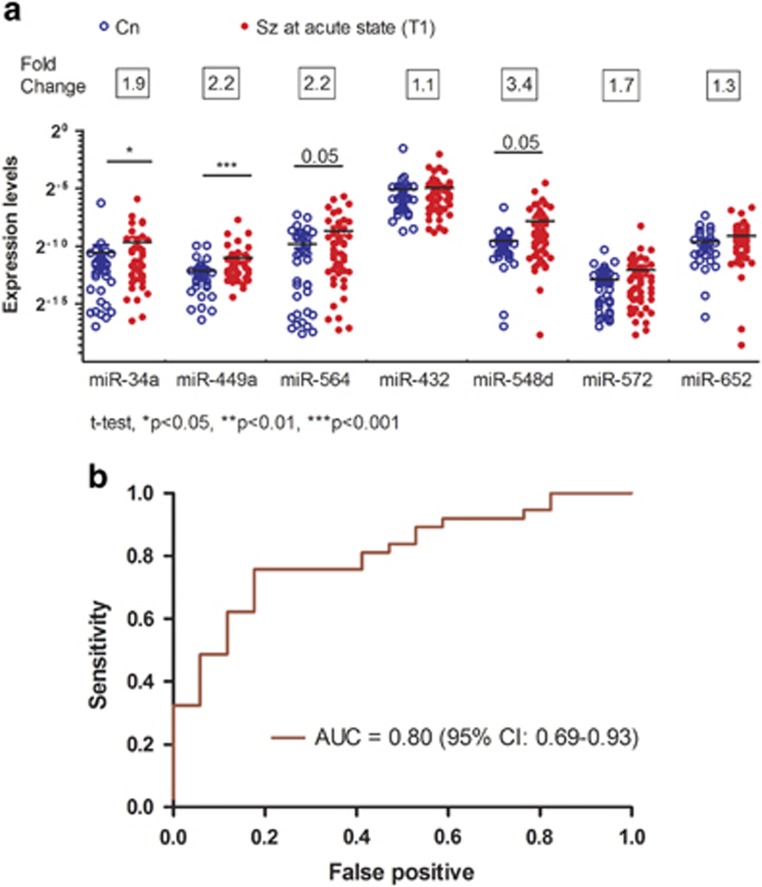
Comparison between people with schizophrenia and controls in peripheral blood cohort. (**a**) The relative expression levels of the seven-miRNA in the peripheral blood cohort of controls (Cn) (*n*=37, hollow circles) and people with schizophrenia (Sz) (*n*=48, solid circles) at acute state (T1), *P<0.05 and ***P<0.001. (**b**) The area under receiver operating characteristic curve (AUC) of the seven-miRNA signature in distinguishing the Sz and Cn.

Before comparing the levels of the miRNAs between baseline and 2-month partial remission, we first examined whether this cohort replicated the discrimination validity of the seven-miRNAs signature. Figure 1 depicts the expression levels of the seven miRNAs for the peripheral sample cohort. Levels of hsa-miR-34a (*t*_75_=2.1, *P*<0.05, fold change=1.9) and hsa-miR449a (*t*_76_=3.5, *P*<0.001, fold change=2.2) were higher in the PBMC from people with schizophrenia compared with controls (Figure 1a). The other two miRNAs, hsa-miR-564 (*t*_79_=1.98, *P*=0.05, fold change=2.2) and hsa-miR-548d (*t*_79_=1.97, *P*=0.05, fold change=3.4), were upregulated in the PBMC with borderline significance level. The global performance of the seven-miRNA signature, as determined by the AUC distinguished people with schizophrenia from controls, with an accuracy of 0.80 (95% confidence interval: 0.69–0.93) (Figure 1b). The sensitivity and specificity at an optimal cut-off were 75.7% and 86.5%, respectively.

**Figure 2:**
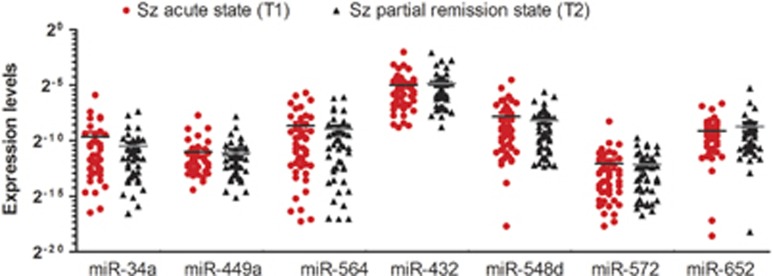
The expression levels of the seven-miRNA in the peripheral blood cohort at acute (T1) and partial remission stage (T2) in people with schizophrenia (Sz, *n*=48). The seven miRNAs were not substantially altered between T1 and T2 of people with schizophrenia.

When we compared the levels of the miRNAs between baseline and 2-month partial remission with significant improvement in clinical symptoms (Supplementary Table S2), we found that peripheral blood levels of the seven miRNAs were not substantially altered between the time points of admission (T1) and discharge (T2) of people with schizophrenia (Figure 2), indicating that the levels of these miRNAs in PBMCs not being altered by the hospitalization in the acute period of 2 months.

### Demographic, tissue collection and pharmacological data of human CNS cohort

In this cohort, people with schizophrenia had a higher suicide rate than their age–gender matched controls (Table 1). The age at onset and DOI of people with schizophrenia were 25.6±9.8 years and 17.5±15.4 years, respectively. The chlorpromazine equivalents of the final recorded doses of the anti-psychotic drugs were 651.8±628.5 mg per day.

Adopting a previous cut-off value for dividing DOI in this postmortem sample cohort,^9^ the people with long DOIs (>22 years, mean age=57 years) and their matched controls (mean age=58.3 years) were older (*P*<0.001) than those with short DOI (<7 years, mean age=30.4 years) and their matched controls (mean age=27.2 years) (Supplementary Table 3).

### miRNAs of human CNS cohort

**Figure 3:**
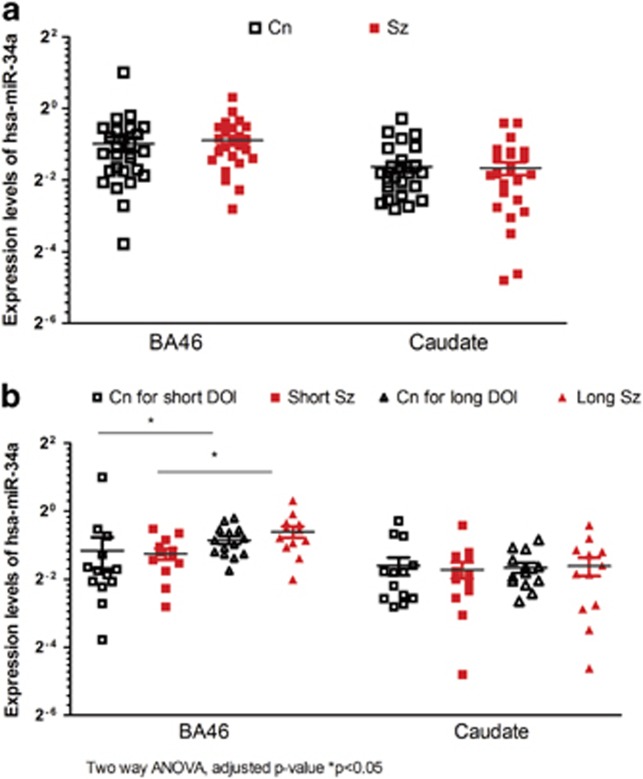
(**a**) Expression levels of hsa-miR-34a in BA46 and caudate of healthy control (Cn, *n*=27) and people with schizophrenia (Sz, *n*=25). (**b**) Expression levels of hsa-miR-34a in BA46 and caudate of non-psychiatric controls (Cn for short DOI, *n*=13; Cn for long DOI, *n*=14) and people with schizophrenia with short (Short Sz, *n*=13, solid square) or long duration of illness (Long Sz, *n*=12, solid triangle) *P < 0.05.

Figure 3 depicts the expression levels of the two miRNAs for the postmortem sample cohort. Compared with controls, there were no significant differences in the expression levels of hsa-miR-34a in either BA46 or caudate putamen from persons with schizophrenia (Figure 3a). In addition, levels of hsa-miR-34a did not differ in BA46 or caudate putamen from subjects with short DOI or long DOI compared with their matched controls (short DOI: *t*_45_=0.6, fold change=0.9; long DOI: *t*_45_=1.2, fold change=1.2) (Figure 3b). By contrast, levels of hsa-miR-34a were higher in BA46 from people with schizophrenia with long DOI compared with those with a short DOI (*t*_45_=2.7, adjusted *P*=0.04, fold change=1.6) and when comparing long and short DOI control groups (*t*_45_=3.0, adjusted *P*=0.02, fold change=1.2) (Figure 3b).

The expression levels of hsa-miR-548d were much lower (17.7 times) than in the PBMC,^17^ which meant we could not reliably measure the expression levels of this miRNA in CNS tissue because of assay sensitivity.

### Age and miRNA expressions in CNS and periphery

**Figure 4:**
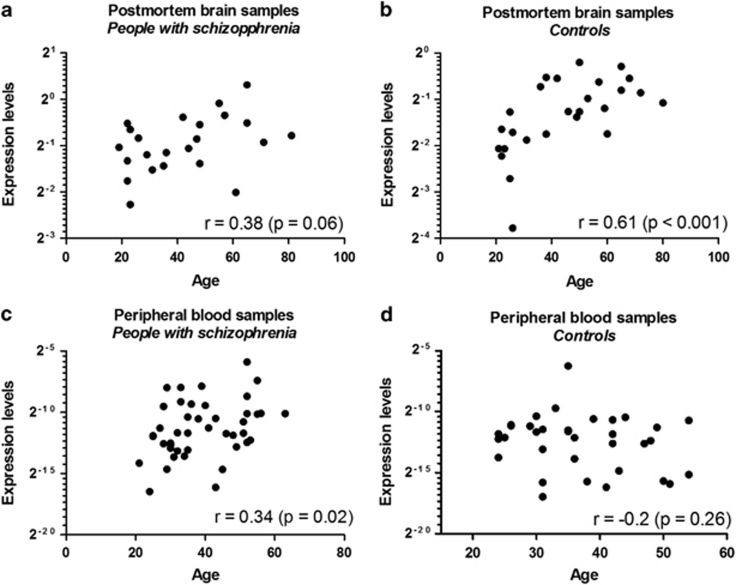
Scatter plot of age and hsa-miR-34a expression levels in BA46 (**a**) of people with schizophrenia (*n*=23) and (**b**) healthy controls (*n*=26) and in PBMCs of (**c**) people with schizophrenia (*n*=43) and (**d**) healthy controls (*n*=34). The correlation coefficient and its *P*-value were indicated by *r* and *p*, respectively.

To further examine whether the expression levels of hsa-miR-34a changed with age, we conducted a series of correlation analyses. In the postmortem brain samples, there was a weak correlation between hsa-miR-34a level and age in people with schizophrenia (*r*=0.31, *P*=0.067, Figure 4a) and a moderate correlation in controls (*r*=0.61, *P*<0.001, Figure 4b). Adjusting for gender had little impact on these correlations in the schizophrenia (*r*=0.32, *P*=0.067) and control (*r*=0.63, *P*=0.002) cases, respectively. In the PBMC samples, there was a weak correlation between hsa-miR-34a level and age in people with schizophrenia (*r*=0.34, *P*=0.02, Figure 4c) but no correlation in controls (*r*=−0.2, *P*=0.26, Figure 4d). Similarly adjusting for gender did not substantially affect these relationships in schizophrenia (*r*=0.34, *P*=0.028) and controls (*r*=−0.3, *P*=0.12). When the associations with hsa-miR-34a level and age were regressed out, hsa-miR-34a still showed upregulation (F_(1,74)_=3.90, *P*=0.05) in people with schizophrenia.

For the results of differential correlations between age and hsa-miR-34a levels in the cases and controls, we further conducted a linear regression analysis of hsa-miR-34a levels with an interaction term between age and disease status (Supplementary Table 4). In the postmortem sample cohort, there were no significant interactions between age and disease status on hsa-miR-34a levels with/without adjustment for gender (*P*=0.15, Supplementary Figures 2A and B). In contrast, the correlation of age with hsa-miR-34a in the periphery sample cohort was affected by disease status for both the model without (*P*=0.02, Supplementary Figure 2C) and with adjustment for gender (*P*=0.01, Supplementary Figure 2D).

## Discussion

To our knowledge, this is the first study to examine the expression changes in seven candidate miRNAs in the PBMC of people with schizophrenia at different clinical time points, and whether the aberrant expression in the PBMC of two selected miRNAs could be replicated in the postmortem brain from an independent sample of people with schizophrenia. Our results revealed that the expressions of the seven miRNAs in PBMC were not affected by the hospitalization of 2 months, which resulted in significant improvement of clinical symptoms. The aberrant expressions seen in the PBMC of two candidate miRNAs (hsa-miR-34a and hsa-miR-548d) were not replicated in the brain samples. However, we found age-dependent increases in hsa-miR-34a expressions in human cortical region (that is, BA46) but not in subcortical region. Furthermore, the correlation between hsa-miR-34a expression level in BA46 and age was much stronger in the controls than in the cases, and the corresponding correlation in PBMCs was only seen in the cases. These findings provide further insights about peripheral miRNAs as potential stable biomarkers for the diagnosis of schizophrenia.

It is of interest to note that the pattern of aberrant expressions of the seven miRNAs in discriminating people with schizophrenia from healthy controls are consistent with our previous finding.^17^ Although the relative expression of only two miRNAs (hsa-miR-34a and hsa-miR-449a) reached statistical significance in the peripheral sample, the remaining four, except hsa-miR-432, tended to be upregulated. Intriguingly, the finding of another peripheral study using a cohort of preterm infants and adults^35^ showed that six (hsa-miR-34a, hsa-miR-449a, hsa-miR-564, hsa-miR-432, hsa-miR-548d and hsa-miR-572) of the seven schizophrenia-associated miRNAs were consistently expressed from infancy to adulthood. Thus, it is possible that the PBMC miRNAs are markers for neurodevelopmental traits. A longitudinal study of a large population at risk of developing schizophrenia is warranted to clarify the status of these markers.

Importantly, the aberrant expressions of the seven miRNAs in the PBMC remained unchanged after ~2 months of hospitalization and treatment. The lack of change in expression of miRNAs following anti-psychotic treatment was reported for some miRNAs. A study examining the plasma levels of nine candidate miRNAs in plasma both at baseline and after 6 weeks of treatment showed expression of hsa-miR-181b, but not hsa-miR-34a and hsa-miR-432, was downregulated following treatment.^7^ In addition, partial support for the lack of change after treatment comes from pharmacological studies, which showed that anti-psychotic drugs altered the expression of <1% of mouse central miRNAs^19^ or those expressed by T-lymphocyte cells.^8^ In the mouse, only three miRNAs (mmu-miR-193, mmu-miR-434-5p and mmu-miR-22) showed altered expression following treatment with haloperidol, olanzapine and clozapine for 7 days.^19^ Likewise, only two miRNAs (hsa-miR-220c-3p and hsa-miR-28-5p) showed altered expression in T-lymphocyte cell lines following 15 days of incubation with chlorpromazine, haloperidol and clozapine.^8^ This suggests that the miRNAs could be traits rather than state-dependent markers and their expressions are not affected by the treatments that resulted in patients being well enough to be discharged from hospital.

While the changes in expression of hsa-miR-34a and hsa-miR-548d seen in the periphery were not present in the two brain regions examined in this study, this does not negate the possibility that the expression changes in miRNAs in the PBMC could be clinically useful biomarkers for schizophrenia. As shown in our previous study hsa-miR-34a and hsa-miR-548d were differentially associated with a variety of neurocognitive tests,^17^ the blood-based miRNA expressions could reflect the brain activity but not necessarily through the dysregulation of miRNAs in the BA46 or caudate putamen of people with schizophrenia. Furthermore, the lack of change in the expression of hsa-miR-34a in BA46 and the caudate putamen from people with schizophrenia are consistent with a previous study on the miRNA profiles in BA46 from people with schizophrenia.^22^ These findings are in disagreement with an earlier study, which reported that miR-34a was upregulated in BA46 of people with schizophrenia.^21^ However, that finding was based on a multiple regression model with adjustment of gender and the pH of postmortem brain, suggesting the discrepant outcomes might be due to differences in the statistical approaches.

Unexpectedly, we found age-related changes in hsa-miR-34a expression in BA46 from both people with schizophrenia and controls. The result in the brain samples was supported by similar findings of increasing expression of miR-34 with age in *Drosophila*, *Caenorhabditis elegans* and human brain.^36, 37, 38^ miR-34 is a markedly conserved miRNA, with orthologues in fly, *C. elegans*, mouse and humans,^39^ therefore it may impact on adult-onset, age-associated events by silencing developmental genes. For example, upregulation of miR-34 in *Drosophila* extends median lifespan and mitigates the neurodegeneration induced by polyglutamine disease protein.^39^ Furthermore, seven target genes of hsa-miR-34a (GREM2, CAMSAP1, TANC2, CALN1, RGMB, FKBP1B and RTN4RL1) are related to neural development and function and were downregulated in human superior frontal gyrus during aging,^38^ suggesting that there may be a functional association between these trends. Nevertheless, a recent study profiling miRNA in BA46 from healthy individuals did not report age-related changes in the expression of hsa-miR-34a.^40^ It is noteworthy that they used a bead-based miRNA microarray platform to quantify hsa-miR-34a,^40^ rather than the qPCR used by the other studies. Hence, the approaches of statistical analysis on the age-related changes in miRNA expression were different between ours and the other two human postmortem brain studies mentioned above.^38, 40^

In the postmortem brain samples, whether or not there is interaction between age and disease status on hsa-miR-34a expression levels warrants further investigation. The sample size of this study is relatively small to draw a conclusion from. Meanwhile, aging may interact with disease status on hsa-miR-34a expression levels in the periphery sample cohort, where stratified analyses showed the correlation of aging with hsa-miR-34a expression levels in the people with schizophrenia only. Although schizophrenia is often conceptualized as a neurodevelopmental disorder,^41^ there is increasing evidence showing people with schizophrenia have accelerated physical aging (with increased and premature medical comorbidity and mortality) in comparison with the general population,^42^ such as dysregulation in inflammation^43^ and oxidative stress markers,^44^ more metabolic symptoms^45^ and shorter telomere.^46^ Our findings in the peripheral samples are likely to support the argument that accelerated changes with aging exist in schizophrenia.

There are some limitations associated with our study. First, most (>90%) of the people with schizophrenia who provided peripheral samples had previous hospitalizations and thus treatment for the illness. Therefore, while the positive symptom scores of patients in partial remission were improved when compared with the acute state, our study may underestimate the effect of anti-psychotic treatment on these changes. Second, for each DOI subgroup of postmortem brain cohort (*n*<15) the sample size was relatively small to detect differences, since the power to detect an effect size lower than 1.3 was <60% in this study. The fold change of expression levels in BA46 between cases and controls were 0.94 and 1.19 for short and long DOIs, respectively. Nevertheless, the age-related changes in expression were detected between short and long DOI group in either the people with schizophrenia (fold change=1.57) or controls (fold change=1.24), suggesting that the lack of change is not due to lack of power. Third, the suicide rate was much higher in people with schizophrenia who had short DOIs. Despite the report that suicide might be associated with miRNA expression changes,^20^ the expression levels of hsa-miR-34a were not different between suicide and non-suicide individuals of BA46 (*t*_23_=0.1, *P*=0.9) and caudate putamen (*t*_24_=0.6, *P*=0.5) in our study (Supplementary Figure 1). In addition, hsa-miR-34a has never been reported to be correlated with suicide.^47^ Finally, there is some heterogeneity between the peripheral and postmortem cohorts used in this study: (i) the major ethnic groups of the cohorts are Han Chinese and Caucasian. Ethnicity could affect the association between miRNA polymorphisms and cancer,^48^ but there is no evidence that ethnicity affects miRNA expressions; (ii) the percentage of males was higher (*χ^2^*_1_=18.2, *P*<0.0001) in the postmortem sample cohort (82.7%) than in the peripheral sample cohort (45.9%) (Table 1). Importantly, hsa-miR-34a in the peripheral sample, but not in postmortem brain cohort, had higher levels of expression in males than in females (*t*_75_=2.0, *P*=0.05, fold change=2.1). Nevertheless, our findings remain similar with or without adjustment for gender.

In conclusion, our results provide novel information regarding the differences in dynamic changes between peripheral and cortical miRNA expressions with clinical course. Peripheral miRNA expressions were not affected by 2 months of hospitalization, and this stability suggests peripheral miRNA might have potential as trait biomarkers for schizophrenia. While the aberrant expressions of two miRNAs in the periphery were not present in the two brain regions studied, this does not negate the possibility that the expression changes in miRNAs in the PBMC could be clinically useful biomarkers for schizophrenia. Furthermore, the association between the miRNA dysregulations, the disease predisposition and aging warrants further investigation. Taken together, this study has provided further insights of the peripheral miRNA as potential stable biomarkers for diagnostic of schizophrenia.

## Figures and Tables

**Table 1:** The demographic of schizophrenia patients and the non-psychiatric controls in the peripheral blood cohort and the postmortem brain cohort

*Variable*	*Peripheral blood cohort*	
	*Schizophrenia (*N=*48)*	*Controls (*N=*37)*
	N *(%)*	N *(%)*
*Gender*		
Female	23 (47.9)	23 (62.2)
Male	25 (52.1)	14 (37.8)

	Mean (s.d.)	Mean (s.d.)
Education (years)^a^	11.3 (2.9)	17.1 (3.5)
Age	40.2 (10.7)	38.0 (10.8)
Age at onset	23.6 (7.0)	—
Duration of illness	16.8 (10.5)	—
Chlorpromazine equivalent (mg daily)	453.3 (276.1)	—

	*Postmortem brain cohort*	
	*Schizophrenia (*N=*25)*	*Controls (*N=*27)*
	N *(%)*	N *(%)*
*Gender*		
Female	4 (16.0)	5 (19.5)
Male	21 (84.0)	22 (81.5)

*Suicide*^b^		
Yes	9 (36.0)	0 (0.0)
No	16 (64.0)	27 (100.0)

	Mean (s.d.)	Mean (s.d.)
Age	43.4 (18.3)	43.3 (18.1)
Age at onset	25.6 (9.8)	—
Duration of illness	17.5 (15.4)	—
PMI (h)	40.4 (12.4)	41.4 (15.2)
Brain pH	6.3 (0.2)	6.3 (0.2)
RIN	8.3 (0.7)	8.5 (0.5)
Chlorpromazine equivalent (mg daily)	651.8 (628.5)	—

Abbreviations: PMI, postmortem interval; RIN, RNA integrity numbers.

a *P*<0.001 based on *t-*test.

b *P*<0.0001 based on Fisher's exact test.
