# Supplementary material for: Aberrant expression of microRNAs as biomarker for schizophrenia: from acute state to partial remission, and from peripheral blood to cortical tissue
Source: Transl Psychiatry. 2016 Jan 19;6(1):e717–. doi: 10.1038/tp.2015.213 (PMC5068884; doi:10.1038/tp.2015.213)
Supplement: Supplementary Information [file tp2015213x1.docx]

**Supplement**

**Supplementary Table 1.** TaqMan MicroRNA Assays (Life Technologies). The list of primers /probes for the seven microRNAs.

**Supplementary Table 2**. The scores of Positive and Negative Syndrome Scale of the peripheral blood cohort of schizophrenia patients at acute (T1) and remission (T2) times

**Supplementary Table 3**. The demographics of the postmortem brain cohort of schizophrenia patients with short (< 7 years) /long duration illness (> 22 years) and the respective control groups.

**Supplementary Table 4**. The results of the linear regression analysis of hsa-miR-34a on age, disease status and the interaction of age and disease status in postmortem brain sample cohort (n = 49) and the peripheral sample cohort (n = 77)

**Supplementary Figure 1. T**he expression levels of hsa-miR-34a in suicide and non-suicide individuals

**Supplementary Figure 2.** The plot of the linear regression of hsa-miR-34a expression levels against age, disease status, interaction of age and disease status in BA46 of postmortem brain cohort (n = 49) A) without and B) with adjustment for gender. Both models showed no interaction (p-value _(interaction term)_ = 0.15) of age and disease status on hsa-miR-34a expression levels. The peripheral sample cohort (n = 77) was subjected to same models, and showed that the disease status effect on the correlation between age and hsa-miR-34a expression levels both C) without (p-value _(interaction term)_ = 0.02) and D) with adjustment (p-value _(interaction term)_ = 0.01) for gender

**Supplementary Table 1.** TaqMan MicroRNA Assays (Life Technologies). The list of primers /probes for the seven microRNAs.

| Assay ID | Assay Type | Availability | Assay Name | Mature microRNA Sequence | miRBase Names | miRBase Accession |
| --- | --- | --- | --- | --- | --- | --- |
| 000426 | Mature miRNA | Inventoried | hsa-miR-34a | UGGCAGUGUCUUAGCUGGUUGU | hsa-miR-34a-5p | MIMAT0000255 |
| 001030 | Mature miRNA | Inventoried | hsa-miR-449 | UGGCAGUGUAUUGUUAGCUGGU | hsa-miR-449a | MIMAT0001541 |
| 001531 | Mature miRNA | Inventoried | hsa-miR-564 | AGGCACGGUGUCAGCAGGC | hsa-miR-564 | MIMAT0003228 |
| 001026 | Mature miRNA | Inventoried | hsa-miR-432 | UCUUGGAGUAGGUCAUUGGGUGG | hsa-miR-432-5p | MIMAT0002814 |
| 001605 | Mature miRNA | Inventoried | hsa-miR-548d | CAAAAACCACAGUUUCUUUUGC | hsa-miR-548d-3p | MIMAT0003323 |
| 001614 | Mature miRNA | Inventoried | hsa-miR-572 | GUCCGCUCGGCGGUGGCCCA | hsa-miR-572 | MIMAT0003237 |
| 002352 | Mature miRNA | Inventoried | hsa-miR-652 | AAUGGCGCCACUAGGGUUGUG | hsa-miR-652-3p | MIMAT0003322 |

**Supplementary Table 2**. The scores of Positive and Negative Syndrome Scale of the peripheral blood cohort of schizophrenia patients at acute (T1) and remission (T2) times

| PANSS | Items | T1 (n = 44) Mean (SD) | T2 (n = 26) Mean (SD) | Paired Change^b^ (n=25) Mean (SD) | P-value ^a^ |
| --- | --- | --- | --- | --- | --- |
| Total score | 30 | 71.1 (19.2) | 61.9 (24.1) | -12.7 (16.0) | p < 0.001 |
| Positive symptoms | 7 | 19.4 (4.3) | 16.0 (6.8) | -4.9 (5.6) | p < 0.001 |
| Negative symptoms | 7 | 18.0 (8.2) | 16.6 (9.3) | -1.8 (6.2) | p = 0.15 |
| General Psychopathology symptoms | 16 | 33.6 (10.4) | 29.3 (10.4) | -6.0 (7.5) | p < 0.001 |
| ^a^Based on paired t-test |  |  |  |  |  |
| ^b^Paired Change = T2 – T1 |  |  |  |  |  |

**Supplementary Table 3**. The demographics of the postmortem brain cohort of schizophrenia patients with short (< 7 years) /long duration illness (> 22 years) and the respective control groups.

|  | Short duration group | |  | Long duration group | |
| --- | --- | --- | --- | --- | --- |
|  | Control  (N = 13) | Schizophrenia  (N = 13) |  | Control  (N = 14) | Schizophrenia  (N = 12) |
|  | **N (%)** | | | | |
| Gender |  |  |  |  |  |
| Male | 10 (76.9) | 11 (84.6) |  | 12 (85.7) | 10 (83.3) |
| Female | 3 (23.1) | 2 (15.4) |  | 2 (14.3) | 2 (16.7) |
| Suicide ^a^ |  |  |  |  |  |
| Yes | 0 (0.0) | 9 (69.2) |  | 0 (0.0) | 0 (0.0) |
| No | 13 (100.0) | 4 (30.8) |  | 14 (100.0) | 12 (100.0) |
|  | **Mean (SD)** | | | | |
| Age ^b^ | 27.2 (6.4) | 30.4 (13.1) |  | 58.3(10.8) | 57 (12.0) |
| Age at onset | - | 26.0 (12.5) |  | - | 25.3 (6.3) |
| Duration of illness ^c^ | - | 4.4 (1.7) |  | - | 31.8 (9.3) |
| PMI ^d^ (hours) | 46.1 (10.9) | 44.2 (13.8) |  | 37.1(17.5) | 36.4 (9.8) |
| Brain pH | 6.3 (0.2) | 6.3 (0.2) |  | 6.4(0.2) | 6.3 (0.2) |
| Chlorpromazine equivalent (mg Daily) | - | 773.8 (796.8) |  | - | 519.6 (364.1) |
| ^a^ p<0.0001 based on Fisher exact test | | | | |  |
| ^b^ p<0.0001 based on analysis of variance | | | | |  |
| ^c^ p<0.0001 based on T test | | | | |  |
| ^d^PMI, postmortem interval | | | | |  |

**Supplementary Table 4**. The results of the linear regression analysis of hsa-miR-34a on age, disease status and the interaction of age and disease status in postmortem brain sample cohort (n = 49) and the peripheral sample cohort (n = 77)

|  | Brain Sample cohort | | |  | Blood sample cohort | | |
| --- | --- | --- | --- | --- | --- | --- | --- |
|  | Standardized Beta | SE^a^ | p-value |  | Standardized Beta | SE | p-value |
| Model 1 |  |  |  |  |  |  |  |
| Intercept | -0.28 | 0.16 | 0.09 |  | -0.26 | 0.17 | 0.13 |
| Age | 0.66 | 0.17 | 0.0002 |  | -0.24 | 0.2 | 0.24 |
| Dis^b^ | 0.57 | 0.24 | 0.02 |  | 0.54 | 0.23 | 0.02 |
| Age x Dis | -0.35 | 0.24 | 0.14 |  | 0.58 | 0.25 | 0.02 |
| Model 2 |  |  |  |  |  |  |  |
| Intercept | -0.30 | 0.17 | 0.09 |  | -0.005 | 0.22 | 0.98 |
| Age | 0.66 | 0.17 | 0.0003 |  | -0.29 | 0.2 | 0.15 |
| Dis | 0.57 | 0.24 | 0.02 |  | 0.50 | 0.22 | 0.03 |
| Gender | 0.09 | 0.33 | 0.79 |  | -0.44 | 0.22 | 0.05 |
| Age x Dis | -0.35 | 0.24 | 0.15 |  | 0.62 | 0.25 | 0.01 |
| ^a^SE: Standard error  ^b^Dis: Disease status | |  |  |  |  |  |  |

**Supplementary Figure 1. T**he expression levels of hsa-miR-34a in suicide and non-suicide individuals

**Supplementary Figure 2.** The plot of the linear regression of hsa-miR-34a expression levels against age, disease status, interaction of age and disease status in BA46 of postmortem brain cohort (n = 49) A) without and B) with adjustment for gender. Both models showed no interaction (p-value _(interaction term)_ = 0.15) of age and disease status on hsa-miR-34a expression levels. The peripheral sample cohort (n = 77) was subjected to same models, and showed that the disease status effect on the correlation between age and hsa-miR-34a expression levels both C) without (p-value _(interaction term)_ = 0.02) and D) with adjustment (p-value _(interaction term)_ = 0.01) for gender

**
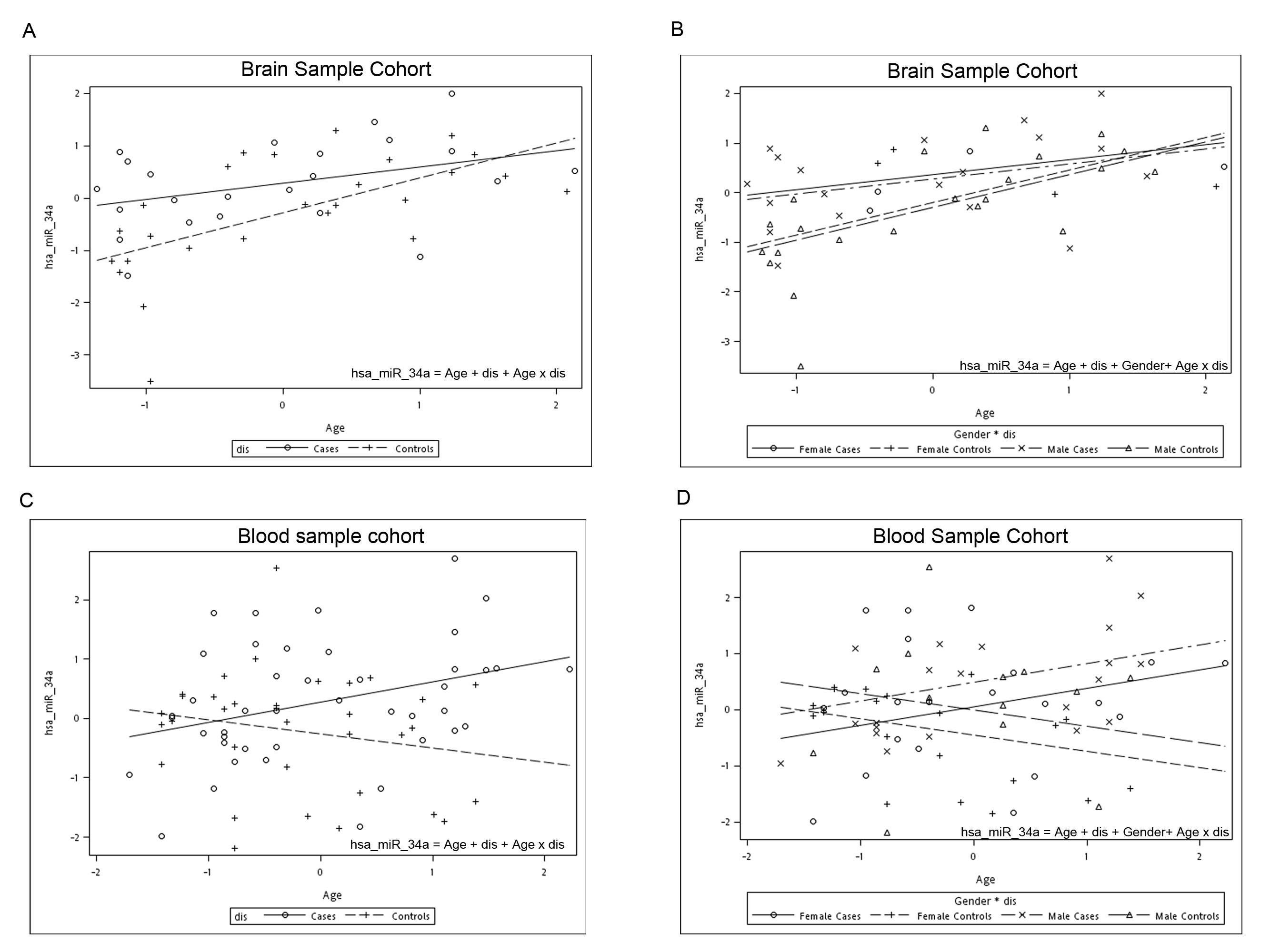
**
